# Comparison of functionality, physical activity, cardiac and respiratory parameters between patients with mood disorders and healthy controls

**DOI:** 10.1192/bjo.2024.758

**Published:** 2024-09-30

**Authors:** İrem Hüzmeli, Nihan Katayıfçı, İrem Görgün, Eren Lekesiz, Mehmet Hanifi Kokaçya

**Affiliations:** Department of Physiotherapy and Rehabilitation, Faculty of Health Sciences, Hatay Mustafa Kemal University, Turkey; Department of Psychiatry, Hatay Mustafa Kemal University Medical School, Hatay, Turkey

**Keywords:** Psychotic mood disorders, functional performance, respiratory function tests, dyspnoea

## Abstract

**Background:**

The cardiorespiratory effect in mental illnesses has recently received much attention. However, the cardiovascular and pulmonary effects of mood disorders have not been clearly demonstrated.

**Aims:**

This study aims to compare individuals with mood disorders and healthy people in terms of exercise capacity, functionality, respiratory muscle strength, pulmonary function, dyspnoea and physical activity level.

**Method:**

This cross-sectional study involved 30 patients with mood disorders and 35 age- and gender-matched healthy individuals. Exercise capacity (6-Minute Walk Test (6MWT), 3-Minute Step Test (3MST)), functionality (vertical jump test, functional reach test), respiratory parameters (respiratory muscle strength, pulmonary function test), dyspnoea (Modified Medical Research Council Dyspnoea Scale) and physical activity level (Short-Form International Physical Activity Questionnaire (IPAQ)) were evaluated.

**Results:**

6MWT results (*P* < 0.001) and functional test scores (vertical jump test, *P* = 0.006; functional reach test, *P* < 0.001) were significantly lower, and heart rate recovery after 3MST (*P* < 0.001) was higher in mood disorder patients. Although patients' respiratory parameters were lower than healthy individuals, only measured and predicted respiratory muscle strength (*P* < 0.001), peak expiratory flow rate litres (*P* < 0.001), forced vital capacity predicted (*P* = 0.010) and forced expiratory volume in 1 s predicted (*P* = 0.002) values were statistically significantly different. Dyspnoea with activities was higher in patients (*P* < 0.001). Patients spent more time sitting (IPAQ, *P* < 0.001), but overall physical activity levels were similar between the two groups (*P* > 0.05).

**Conclusions:**

Patients with mood disorders had decreased exercise capacity and pulmonary function, lower functionality scores and respiratory muscle strength, and increased dyspnoea. Exercise-based rehabilitation protocols are recommended for the management of risk factors affecting the mood disorder patients' cardiorespiratory status.

A person's mood is described as a pervasive and enduring feeling tone that affects almost all facets of their conduct in the outside world. Affective disorders, or mood disorders, are characterised by major emotional disruptions (e.g. extreme lows like depression or highs like hypomania or mania). These are widespread psychiatric conditions that raise morbidity and mortality rates.^1,2^ Mental disorders, including affective disorders, contribute to the global burden of disease, and advanced mental illnesses lead to the risk of premature death.^1^

Although genetic factors and pathophysiological mechanisms contribute to mood disorders, treatment-related factors and unhealthy lifestyle habits play a prominent role in the development of cardiovascular disease and respiratory disease in individuals with mental illness, including mood disorders.^3–5^ Mood disorders, such as depression, have also been shown to have a negative effect on physical activity, well-being and low cardiorespiratory fitness.^6–8^ Cardiorespiratory fitness plays an important role in determining mortality in many diseases, such as cardiovascular and respiratory diseases.^6–8^ In this respect, it is clinically important to investigate cardiorespiratory suitability in individuals with mood disorders.^9^ Impaired cardiorespiratory fitness has been associated with psychiatric symptoms and cognitive deficits.^10^ As physical inactivity and fitness are closely related, weak respiratory muscle strength is correlated with normal pulmonary function and increased dyspnoea in patients with major depression. Changes in respiratory muscle strength affect pulmonary function tests and decrease vital capacity.^11,12^

Cardiorespiratory impact in mental diseases has received much attention in the literature. However, cardiovascular and pulmonary effects in mood disorders have not been sufficiently investigated. In an attempt to overcome this limitation, the current study was designed to compare the exercise capacity, functionality, respiratory muscle strength, pulmonary function, dyspnoea and physical activity level between patients with mood disorders and healthy controls. Also, we hypothesised that patients with mood disorders have a significantly higher burden of respiratory and cardiovascular symptoms compared with controls.

## Method

This cross-sectional study was carried out with patients with a mood disorder and a healthy control group. The patient group consisted of 30 patients with diagnosis of mood disorder who applied to the Hatay Mustafa Kemal University Research and Practice Hospital, Mental Health and Diseases Out-patient Clinic, whereas the control group included 35 healthy individuals. The groups were matched in terms of age, gender and body mass index. The authors assert that all procedures contributing to this work comply with the ethical standards of the relevant national and institutional committees on human experimentation and with the Helsinki Declaration of 1975, as revised in 2008. All procedures involving human patients were approved by the Hatay Mustafa Kemal University Non-Interventional Ethics Committee (approval number: 12.01.2023/02-20). The study was registered with Clinicaltrials.gov (registration number: NCT06181123). Before participation, every participant provided signed informed consent documents. The study was carried out between May and November 2023.

Inclusion criteria for the patient group were as follows: being over 18 years of age and diagnosed with a mood disorder. Patients diagnosed with bipolar disorder type 1, bipolar disorder type 2 or major depression according to the DSM-V, who were admitted to hospital and demonstrated the ability to concentrate for at least 30 min, were evaluated by a trained psychiatrist during a semi-structured interview. Patients who were admitted to the emergency psychiatric service were not included in the study.

The control group comprised volunteers who had undergone a general physical examination in the past 6 months and reported being free of significant cardiovascular, respiratory, neuromuscular and endocrine disorders that precluded safe participation.

Exclusion criteria were individuals with cardiovascular, neuromuscular or endocrine disorders; pregnant women; people under 18 years old and disabled individuals (patient file records were scanned).

### Outcomes

The following were evaluated to make comparisons between the two groups: demographic data, clinical characteristics, exercise capacity (6-Minute Walk Test (6MWT), 3-Minute Step Test (3MST)), functionality (vertical jump test, functional reach test), respiratory parameters (respiratory muscle strength, pulmonary function test, Modified Medical Research Council Dyspnoea Scale (mMRC)) and physical activity level (Short-Form International Physical Activity Questionnaire (IPAQ)).

The primary outcome measure of the study was exercise capacity score. The secondary outcome measures were functionality, respiratory parameters and physical activity level scores.

The exercise capacity was assessed with the 6MWT. The patients were allowed to rest for at least 30 min before starting the test, which was performed according to American Thoracic Society criteria. Vital signs, fatigue and dyspnoea perception were recorded before and after the test. The participants were asked to walk as fast as they could without running in a 30 m straight corridor for 6 min, at their own walking pace.^13^

The 3MST is another assessment tool of exercise capacity. In this test, the person ascends and descends the 30.5 cm step with the rhythm of the metronome set at 96 beats/min for 3 min. At the end of the test, the person sits and after 5 s, and their heart rate is recorded.^14^

The vertical jump test is used to evaluate performance characteristics. The person to be tested lies on a flat platform as far as they can with one arm, placing equal weight on both extremities, and is marked. Then, the person jumps and the last point they can reach by jumping is marked. Applying these procedures, the difference between the two marked points was recorded.^15^

In the functional reaching test, another functionality test, the individual approaches the wall in a sideways position with shoulder facing the wall. Then, the individual opens their feet at shoulder level and clasps their hand, bringing it to a 90-degree flexion position. The part where the third metacarpal bone of the hand meets the wall is marked. The participant is asked to lie forward as much as possible without their heels breaking contact with the ground and without taking a step. Then, the distance between this start and end point is recorded.^16^

Respiratory muscle strength was evaluated with a portable, electronic oral pressure measurement device (MicroRPM, Micro Medical England), according to the American Thoracic Society and European Respiratory Society criteria. Respiratory muscle strength was evaluated with an oral pressure device that gives maximal inspiratory pressure (MIP) and maximal expiratory pressure (MEP) results. If the difference was greater than 5% or 5 cm H_2_O between the two best measured values, the measurement was repeated, and the best values for the analyses were kept.^17^

Pulmonary function tests were performed with a portable spirometer (SPIROBANK II USA). Forced vital capacity (FVC), forced expiratory volume in 1 s (FEV_1_), the ratio of FEV_1_ to FVC (FEV_1_/FVC), peak expiratory flow rate (PEF) and forced mid-expiratory flow rate (FEF_25–75%_) were recorded. The test was performed in the sitting position. The best of the three manoeuvres that were technically acceptable and had 95% agreement with each other was selected for statistical analysis.^18^

The mMRC rates the dyspnoea that may occur during daily activities: ‘0’ means no dyspnoea, only with strenuous exercise, and ‘4’ means dyspnoea even with daily activities.^19^

The physical activity level was evaluated with the short form of the IPAQ. The IPAQ consists of seven questions in total. The indices are calculated by adding the frequency (days) and duration (min) of vigorous activity, moderate activity and walking activities in the past 7 days, and sitting time.^20^

### Statistical analyses

Statistical analyses were conducted with IBM SPSS Statistics 25.0 package for Windows (IBM SPSS, Chicago, USA; https://www.ibm.com/support/pages/downloading-ibm-spss-statistics-25).

#### Sample analysis

The sample size was determined by the G*Power software (G*Power, Version 3.1.9.4 for Windows, Franz Faul, Universität Kiel, Germany; https://www.psychologie.hhu.de/arbeitsgruppen/allgemeine-psychologie-und-arbeitspsychologie/gpower). Considering the 6MWT results of a similar study^9^ (590.8 ± 112.6 *v.* 704.2 ± 94.3 m), the ideal sample size was calculated as at least 39 participants, with an effect size of *d* = 1.091919, α error probability of 0.05 and power (1 − β error probability) of 0.95. Therefore, 65 participants were included in the study.

Normality was checked by with visual (histogram and graphs) and analytical methods (Shapiro–Wilk tests). Descriptive statistics included mean values and s.d. for normally distributed variables, and number and percentage values for categorical variables (e.g. region). Independent sample *t*-tests (Student *t*-test) were used to compare the normally distributed variables of the patient group and the healthy group. The difference between both groups was shown with mean ± s.d. and 95% confidence intervals. Mann–Whitney *U*-test was performed for the variables that do not comply with normal distribution, and the difference between two groups was shown in median values (quartiles 25–75%). *P*-values <0.05 were considered as significant.

## Results

**Fig. 1:**
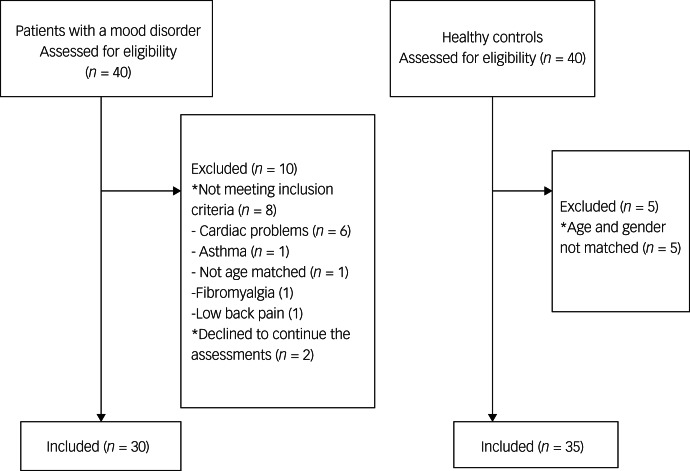
Flow diagram of patients with mood disorders and healthy controls.

From May to November 2023, 40 patients with mood disorders were enrolled in the study. Of those, eight did not meet the inclusion criteria. During the study, two more participants did not want to continue the study, making the final number of 30 participants in the analyses. For the control group, 40 healthy controls were asked to participate, and 35 of them were included in the analyses, as five participants were excluded because they could not be age- and gender-matched (Fig. 1).

Age and gender were matched between the patients and the healthy controls, and demographic and clinical features were examined (Table 1). Statistical difference were found between the groups in the following parameters: current smoker (31.4 and 60%), employed (94.3 and 66.7%), senior education (62.9 and 16.7%), alcohol drinker (11.4 and 20%), dyspnoea (0 and 20%) and sleeping support (0 and 50%) (*P* < 0.05). There were no more statistically meaningful differences in gender, age, weight, height, body mass index and family history (*P* > 0.05). The rate of patients with comorbidities was 10%. Perception of chest pain was present in 43.3% of patients.

**Table 1:** Clinical and demographic characteristics of the participants

	Control group, *n* = 35 Mean ± s.d./median (IQR)	Patient group, *n* = 30 Mean ± s.d./median (IQR)	Mean difference (95% CI)	*P-*value
Age (years)	42.08 ± 9.06	40.16 ± 8.78	−1.91 (−6.36 to 2.54)	0.391^a^
Male/female, *n* (%)	17 (48.6)/18 (51.4)	20 (66.7)/10 (33.3)		0.209^c^
Weight, kg	72.68 ± 12.46	74.96 ± 12.43	3.21 (−4.17 to 8.72)	0.303^a^
Height, m	1.69 ± 0.75	1.68 ± 0.77	−0.011 (−0.03 to 0.306)	0.952^a^
BMI, kg/m^2^	25.16 ± 3.16	26.38 ± 3.64	1.21 (−0.46 to 2.90)	0.188^a^
Normal/overweight/obese, *n* (%)	17 (48.6)/17 (48.6)/1 (2.9)	10 (33.3)/15 (50)/3 (10)		0.205^c^
Education, *n* (%)				
Literate	0 (0)	1 (3.3)		
Primary education or less	6 (17.1)	13 (43.3)		
Secondary education	2 (5.7)	7 (23.3)		**0**.002^c^*
High school education	5 (14.3)	4 (13.3)		
Senior education	22 (62.9)	5 (16.7)		
Smoking, *n* (%)				
Current	11 (31.4)	18 (60)		
Ex-smoker	1 (2.9)	8 (26.7)		**<0**.001^c^*
Non-smoker	23 (65.7)	4 (13.3)		
Packet years	0 (0–0)	20 (10–27.75)		**<0**.001^b^*
Comorbidities, *n* (%)				
Yes/no	Not available	3 (10)/27 (90)		
Family history, *n* (%)				
Yes/no	13 (37.1)/22 (62.9)	8 (26.7)/22 (73.3)		0.432^c^
Occupation, *n* (%)				**0**.016^c^*
Retired	0 (0)	1 (3.3)		
Employed	33 (94.3)	20 (66.7)		
Housewife	1 (2.9)	5 (16.7)		
Unemployed	1 (2.9)	4 (13.3)		
Alcohol consumption, *n* (%)				
Yes/no/ex-drinker	4 (11.4)/31 (88.6)/0 (0)	6 (20)/16 (53.3)/8 (26.6)		**0**.014^c^*
Dyspnoea, *n* (%)				
Yes/no/rarely	0 (0)/31 (88.6)/4 (11.4)	6 (20)/9 (30)/15 (50)		**<0**.001^c^*
Resting dyspnoea, MBS (0–10)	0 (0–0)	0 (0–0.25)	402.50	**0**.003^b^*
Activity dyspnoea, MBS (0–10)	0 (0–0)	3 (0–4)	244.50	**<0**.001^b^*
Chest pain, *n* (%)				
Yes/no/rarely	Not available	13 (43.3)/4 (13.3)/13 (43.3)		
Sleeping support, *n* (%)				
Yes/no	0 (0)/35 (100)	15 (50)/15 (50)		**<0**.001^c^*
mMRC score, *n* (%)				
0	32 (91.4)	0 (0)		
1	2 (5.7)	9 (30)		**<0**.001^c^*
2	1 (2.9)	15 (50)		
3	0 (0)	4 (13.3)		
4	0 (0)	2 (6.7)		

IQR, interquartile range; BMI, body mass index; MBS, Modified Borg Scale; mMRC, Modified Medical Research Council.

*P < 0.05 indicates a statistically significant difference.

a. Normally distributed variables were compared using the Independent Sample *t*-test.

b. Non-normally distributed variables were compared using the Mann-Whitney *U*-test.

c. Categorical variables were compared between groups using the *χ*²-test.

In the mood disorder group, 53.33% of the individuals were diagnosed with bipolar disorder, and 46.67% were diagnosed with major depressive disorder (MDD). Among the patients, 15% were using antipsychotic medication, 30% were using antidepressants and 55% were using both antidepressants and antipsychotics.

### Exercise capacity

Table 2 compares exercise capacity, functionality, respiratory parameters and physical activity in patients with mood disorders and healthy controls. The 6MWT distance and predicted 6MWT score were lower in patients than healthy controls. There was a statistical significance in the differences in resting heart rate, heart rate peak, heart rate recovery, Δ systolic blood pressure, Δ diastolic blood pressure, resting respiratory rate, Δ fatigue at resting, Δ muscle fatigue, Δ dyspnoea and 6MWT completion rate (those who completed the test on time) between the groups. Significant differences were found between the groups in 3MST heart rate scores, 3MST resting diastolic blood pressure, Δ3MST dyspnoea, 3MST SpO_2_ at resting, Δ3MST fatigue and 3MST completion rate scores (*P* < 0.05).

**Table 2:** Comparison of exercise capacity, functionality, respiratory parameters and physical activity in patients with mood disorders and healthy controls

Characteristics	Control group, mean ± s.d./median (IQR)	Patient group, mean ± s.d./median (IQR)	Mean difference (95% CI/U)	*P-*value
Exercise capacity				
6MWT, m	599.04 ± 58.32	383.00 ± 109.36	−216.04 (−258.64 to −173.44)	**<0**.001^a^*
6MWT, % predicted	100.26 (95.33–105.79)	66.45 (81.35–51.84)	16	**<0**.001^b^*
Heart rate resting beats/min	84 (78–88)	87.5 (81.5–89.25)	363	**0**.033^b^*
Heart rate peak	98 (95–110)	108 (100–121.50)	315	**0**.006^b^*
Heart rate recovery	90 (85–95)	97 (94–104)	200	**<0**.001^b^*
SBP resting, mmHg	100 (91–120)	100 (100–110)	499.50	0.728^b^
ΔSBP	10 (0–20)	0 (0–10)	283	**0**.001^b^*
DBP resting, mmHg	80 (80–85)	80 (80–82.50)	502	0.701^b^
ΔDBP, mmHg	0 (0–0)	0 (0–10)	337.50	**0**.006^b^*
Respiratory rate resting, breaths/min	16 (12–20)	12 (12–16)	370	**0**.028^b^*
Δ Respiratory rate	4 (2–4)	4 (0–4)	414	0.094^b^
SpO_2_ resting	90 (87–98)	89 (86–96)	422.50	0.176^b^
ΔSpO_2_	1 (−1–3)	2 (1–4)	396	0.086^b^
Fatigue resting (0–10)	0 (0–0)	0 (0–0)	488	0.247^b^
Δ Fatigue resting	0 (0–0)	0.5 (0–1.25)	340	**0**.003^b^*
Muscle fatigue resting (0–10)	0 (0–0)	0 (0–0)	507.50	0.280^b^
Δ Muscle fatigue	0 (0–1)	1 (0–1)	379.50	**0**.033^b^*
Dyspnoea, 0–10 (resting)	0 (0–0)	0 (0–0)	490	0.124^b^
Δ Dyspnoea	0 (0–0)	0 (0–2)	352.50	**0**.005^b^*
Finished the 6MWT, yes/no, *n* (%)	35 (100)/0 (0)	19 (63.3)/11 (36.7)		**<0**.001^c^*
3MST heart rate, resting pulse/min	85 (79–89)	89 (87–90.25)	281	**0**.001^b^*
3MST, 30 s later	94.51 ± 13.25	121.93 ± 15.49	27.41 (20.29−34.54)	**<0**.001^a^*
3MST 1 min later	90 (82–95)	99 (97–100)	85.00	**0**.001^b^*
Δ3MST heart rate	5.45 ± 5.16	22.80 ± 2.02	17.34 (13.05–21.62)	**<0**.001^a^*
3MST resting SBP, mmHg	100 (100–120)	100 (100–110)	461	**0**.379^b^*
Δ3MST SBP	10 (0–10)	10 (0–10)	484	0.533^b^
3MST resting DBP, mmHg	80 (80–70)	80 (80–90)	324	**0**.020^b^*
Δ3MST DBP	0 (0–10)	0 (0–0)	499	0.706^b^
Respiratory rate, resting breaths/min	16 (12–20)	16 (12–16)	466.50	0.414^b^
Δ3MST respiratory rate	4 (4–4)	4 (0–4)	405.50	0.077^b^
3MST SpO_2_, resting	89 (87–98)	87 (86–93.25)	284.50	**0**.001^b^*
Δ3MST SpO_2_	0.34 ± 3.76	1.90 ± 0.50	1.55 (−0.10 to 3.21)	0.066^a^
3MST fatigue (0–10)	0 (0–0)	0 (0–0)	522.50	0.912^b^
Δ3MST fatigue	0 (0–0)	0 (0–2)	352.50	**0**.005^b^*
3MST dyspnoea (0–10)	0 (0–0)	0 (0–0)	525	>0.001^b^
Δ3MST dyspnoea	0 (0–1)	1 (0.75–1.25)	318.50	**0**.003^b^*
3MST muscle fatigue (0–10)	0 (0–0)	0 (0–0)	472.50	0.057^b^
Δ3MST muscle fatigue	1 (0–2)	1 (0–2)	464.50	0.399^b^
Finished the 3MST, yes/no, *n* (%)	35 (100)/0 (0)	16 (53.3)/14 (46.7)		**<0**.001^c^*
3MST, steps/3 min	88 (75–94)	70 (44–80)	193	**<0**.001^b^*
Functionality				
Vertical jump test, cm	25 (23–27)	21 (19–25)	113.50	**0**.006^b^*
Functional reach test, cm	28 (23–36)	17 (15–20.25)	−6.95 (−9.30 to 4.59)	**<0**.001^b^*
Respiratory parameters				
FEV_1_ litres	3.11 (2.50–3.75)	3.47 (2.44–4.01)	455	0.357^b^
FEV_1_, % predicted	89.45 ± 13.67	75.91 ± 20.44	13.54 (−22.06 to −5.03)	**0**.002^a^*
FVC litres	3.80 (3.01–4.43)	3.51 (4.10–2.96)	466.50	0.301^b^
FVC, % predicted	91 (80–107)	78 (61–80)	201.50	**<0**.001^b^*
FEV_1_/FVC, %	77.20 (73–83.50)	88.70 (70–93.30)	465.50	0.433^b^
FEF_25–75%_ litres	3.07 (2.34–4.18)	3.31 (2.73–3.64)	490.50	0.650^b^
FEF_25–75%_ predicted	77.85 ± 18.14	70.11 ± 16.46	−7.73 (−16.38 to 0.90)	0.079^a^
PEF litres	6.21 (4.34–8.53)	4.09 (3.50–4.83)	236	**<0**.001^b^*
PEF % predicted	77 (63–102)	73 (97.50–74.50)	381	0.058^b^
MIP cm H_2_O	78 (69–98)	71 (64.50–77)	351	**0**.022^b^*
MIP % predicted	86.45 (71.94–102.98)	68.85 (64.68–85.93)	292	**0**.002^b^*
MEP, cm H_2_O	95 (80–109)	66.50 (64–83.25)	175	**<0**.001^b^*
MEP predicted %	87.76 (69.10–106.04)	55.71 (46.79–65.00)	144	**0**.001^b^*
Physical activity				
IPAQ, total MET min/week calculated	622 (2417–346)	582 (435–977)	470	0.469^b^
IPAQ, MET min/week vigorous activity	0 (0–560)	0 (0–0)	452	0.169^b^
IPAQ, MET min/week moderate activity	0 (0–560)	160 (0–480)	489	0.620^b^
IPAQ, MET min/week walking	462 (264–693)	396 (288.75–510.75)	501	0.752^b^
IPAQ, sitting (min per day)	1260 (240–2520)	4725 (3780–6363)	105	**<0**.001^b^*

IQR, interquartile range; 6MWT, 6-Minute Walk Test; SBP, systolic blood pressure; Δ, difference between after the test and 1 min later; DBP, diastolic blood pressure; SpO_2_, oxygen saturation; 3MST, 3-Minute Step Test; FEV_1_, forced expiratory volume in 1 s, FVC, forced vital capacity; FEF_25–75%_, forced expiratory flow at 25–75% of the pulmonary volume; PEF, peak expiratory flow; MIP, maximal inspiratory pressure; MEP, maximal expiratory pressure; IPAQ, International Physical Activity Questionnaire; MET, metabolic equivalent.

*P < 0.05 indicates a statistically significant difference.

a. Normally distributed variables were compared using the Independent Sample *t*-test.

b. Non-normally distributed variables were compared using the Mann-Whitney *U*-test.

c. Categorical variables were compared between groups using the *χ*^2^-test.

### Functionality

The analysis showed a statistically significant difference in vertical jump and functional reach test between patients with mood disorders and healthy controls (*P* < 0.05). The scores for two functionality tests were higher in healthy controls than patients (Table 2).

### Respiratory parameters

Respiratory muscle strength and pulmonary function values are shown in Table 2. The patients had lower predicted FEV_1_, predicted FVC, measured and predicted PEF, measured and predicted MIP and MEP values than healthy controls (*P* < 0.05). There was no statistically significant difference in FEV_1_, FEV_1_/FVC, FEF_25–75%_ and PEF predicted scores between the two groups.

Resting and activity dyspnoea scores (0–10) were higher in the patient group (*P* < 0.05) (Table 1). The proportion of those who received two points according to the MMRC was higher than those who received other scores and was statistically significant between the groups (*P* < 0.005) (Table 1).

### Physical activity

There were similar results between the groups in terms of total IPAQ score and IPAQ vigorous activity, moderate activity and walking scores (*P* > 0.05). However, sitting sore was higher in patients than controls (*P* < 0.05).

## Discussion

The present study aimed to compare various parameters related to exercise capacity, pulmonary function and respiratory muscle strength between individuals with mood disorders and healthy controls. Our findings reveal significant impairments in exercise capacity, functionality and pulmonary function among patients with mood disorders. Additionally, weakened inspiratory and expiratory muscle strength was observed in this group, indicating potential respiratory muscle dysfunction. Dyspnoea in activities of daily living was increased in patients compared with healthy controls. Although physical activity levels were similar, sitting time was longer in patients with mood disorders.

Exercise intolerance, a common feature in cardiopulmonary diseases, significantly affects quality of life by reducing the efficiency of oxygen delivery to working muscles during physical activities.^21^ Notably, individuals with severe mental illnesses, including mood disorders, exhibit poor cardiorespiratory fitness, placing them at higher risk for cardiovascular diseases and mortality. This study further underscores the importance of evaluating cardiorespiratory suitability in individuals with mood disorders, to mitigate associated health risk.^5^ Similarly, patients with mood disorders with no comorbidities had lower 6MWT distance and higher 3MST heart rate recovery than healthy controls in our study. Heart rate intervals depend on respiratory, thermoregulation and baroreflex mechanisms, and constantly change physiologically.^22,23^ Heart rate variability (HRV) reflects the variability in time intervals between consecutive heartbeats.^21^ A previous study showed reduced HRV in bipolar disorders,^24^ whereas others reported an inverse correlation between the severity of bipolar disorder illness and HRV.^25,26^ Although HRV was not measured in the present study, statistically significant differences were found in heart rate recovery and heart rate between groups after the 6MWT and 3MST. The difference in heart rate recovery between the groups may be associated with autonomic nervous system activation or physical activity level. Future studies should investigate the relationship of exercise capacity, daily activities and HRV in mood disorders.

Researchers observed impaired lung function in individuals with depressive illnesses, with a greater impact noted in those diagnosed with MDD.^27^ In contrast, another study discovered an association between MDD and reduced FEV_1_, although no significant difference in lung function was observed when comparing patients with MDD and the control group.^28^ Similarly, there was a decrease in lung function, especially in predicted FEV_1_, predicted FVC and PEF values, in the current study. Afreen et al hypothesised that the diminished psychomotor activity combined with reduced respiratory muscle strength in depressive illness might offer an explanation for the observable effects of depression on impaired lung function in patients with MDD.^29^ Low pulmonary function values may be related to cigarette consumption.^30^ Most of the patients with mood disorders were current smokers, and the median cigarette consumption was 20 pack-years in the patient group in the present study, which may have affected pulmonary function. Contrary to Carrol et al,^28^ but similar to Afreen et al,^29^ reduced psychomotor activity with smoking history may impair respiratory function and strength in patients with mood disorders. However, changes in respiratory function between mood disorders (e.g. bipolar disorder versus MDD) have not been investigated in the current study. Further studies on pulmonary functions in different mood disorders are recommended.

The decrease in pulmonary function was associated with the decrease in respiratory muscle strength.^29^ Similarly, the decrease in inspiratory and expiratory muscle strength were statistically different in the patient group compared with the control group in the current study. In a case-series study with different psychiatric disorders, minimal decrease in pulmonary function test parameters compared with the predicted values, and a decrease in the inspiratory and expiratory muscle strength of patients compared with the predicted values, were observed. According to the study, mean difference of measured and predicted respiratory muscle strength was −29.33 cm H_2_O for MIP and −44.33 cm H_2_O for MEP.^31^ Contrary to this, the mean rank was −10.77 cm H_2_O for MIP and −22.77 cm H_2_O for MEP in our study. Since the current study adopted an experimental design and included more participants (30 compared with five), the current findings offer a more reliable pattern. More research is essential to validate these findings and establish the cause-and-effect correlation between different mood disorders and respiratory functions.

Dyspnoea is one of the most important symptoms of respiratory problems.^32^ Mood disorders such as anxiety/depression are closely associated with dyspnoea. According to a previous study in which mood changes were created in a laboratory in healthy individuals, dyspnoea may vary depending on the mood; lower dyspnoea was observed in those with a positive mood.^33^ Mood variability and dyspnoea affect daily life and reduce active performance. Validating these findings, functional reach and vertical jump test scores were lower in patients with MDD than healthy controls in our study. Even with normal lung function, research suggests that depressive states amplify the perception of exertional dyspnoea.^11,32,34^ Exertional dyspnoea scores were higher after the 6MWT and 3MST, and dyspnoea with activity was different between the groups according to the Modified Borg Scale (0–10) in the current study. The mMRC scores of half of the patients were level 2, which suggests a shortness of breath when walking fast on a flat road. Thus walking, which is one of the activities of daily living, was also affected in patients with mood disorders. Increased exertional dyspnoea indicates decreased cardiorespiratory fitness, and mood disorders might trigger this condition.

Impaired physical abilities affect motor function and daily living activities. Although daily living activities were not examined in this study, all participants showed low physical activity levels. Also, exercise capacity and functional test scores were lower in patients with mood disorders than healthy controls. Smoking has a negative effect on vital organs required for physical activity, such as the brain, nerve tissues and heart, so physical abilities and physical fitness can be affected.^35^ Physiological theories link physical activity to mental health by comparing increased synaptic transmission of monoamines during exercise to antidepressant effects, but this explanation oversimplifies the effect of antidepressants. Another theory suggests that physical activity prompts the release of endogenous opioids, like beta-endorphin, potentially calming the nervous system and enhancing post-exercise mood.^36^ According to these theories and the results of our study, personalised exercise programmes, including respiratory muscle strength and aerobic exercise, are recommended for individuals diagnosed with mood disorders.

Assessment of the physical health in patients with bipolar disorder is an important component in preventing associated comorbidities and properly managing the disease.^37^ Therefore, obesity and cardiovascular risk factors need to be screened regularly. Physical inactivity, alcohol consumption, cigarette consumption, pharmacological treatment and blood lipid levels are important parameters for physical health. An annual physical examination was performed in only 64% of patients receiving mood stabilisers. Additionally, only a minority of patients received health promotion advice (smoking cessation, referral to physical activity, healthy diet, weight loss).^38,39^ In our study, the rate of overweight and obese individuals (60%) was higher in the patient group, and the rate of comorbidities (10%) was low. This may be related to the young age of the patients (40.16 ± 8.78 years). However, impaired respiratory parameters compared with healthy controls, and low physical capacity results may be caused a risk of cardiovascular disease in the future. The findings of our study support the importance of physical activity and lung health, which cause a significant portion of the risk of cardiovascular death in patients with mood disorders. In light of these findings, exercise-based rehabilitation protocols are suggested within the scope of preventive health recommendations for respiratory and cardiac problems that may occur in the future.

Finally, although our study sheds light on the respiratory and cardiovascular implications of mood disorders, certain limitations must be acknowledged. Future research should explore peripheral muscle strength, daily living activities and HRV to provide a comprehensive understanding of the multifaceted relationship between mood disorders and physical health.

The current study is the first to show the change of respiratory parameters in mood disorders, and their effects on functionality. It is also one of the few studies to show changes in cardiac and respiratory system parameters in patients diagnosed with mood disorders, using various evaluation parameters. Further studies are needed to show the changes in daily living and well-being in different mood disorders.

In conclusion, our study underscores the significant impact of mood disorders on exercise capacity, pulmonary function and respiratory muscle strength. These findings highlight the importance of incorporating exercise-based rehabilitation interventions alongside traditional medical treatments, to improve the cardiorespiratory status and overall well-being of individuals with mood disorders.

## Data Availability

The data that support the findings of this study are available from the corresponding author, İ.H., upon reasonable request.
